# Community Perception on Trypanosomosis, Parasitological, and Entomological Studies in Two Selected Districts of South Omo Zone, Ethiopia

**DOI:** 10.1155/2021/8439698

**Published:** 2021-12-24

**Authors:** Solomon Mekuria, Tesfu K. Mekonnen, Nigatu Kebede

**Affiliations:** ^1^Addis Ababa University Aklilu Lemma Institute of Patho-biology, Addis Ababa, Ethiopia; ^2^Hawassa University Faculty of Veterinary Medicine, Hawassa, Ethiopia

## Abstract

Participatory investigation and trypanosomosis prevalence studied during April 2019 and March 2020 in two selected districts of South Omo, Ethiopia. The study site is located in the gridline of 04.90 to 5.60^o^N and 35.80 to 36.90^0^ E. Twelve community groups are employed. A cross-sectional study design and 288 animals bled and examined a wet film prepared from the buffy coat. Sixty NGU traps baited with acetone and cow urine were deployed for 48 hrs to estimate the apparent density. Data generated from focus group discussion and trypanosomosis prevalence analyzed using an appropriate statistical package. Proportional piling showed that cattle, goats, and sheep were proportionally dominant with a high median score of 32(14–40), 26(12–33), and 21(5–23), respectively; trypanosomosis ranked first with a proportional median score of 24(13–26) followed by contagious bovine/caprine pleuropneumonia with a proportional median score of 23(19–26) among others. Community unanimously agreed that (*W* = 0.9) trypanosomosis affects their socioeconomic status and was able to describe clinical signs with significant (*p* < 0.05) agreement. Tsetse fly (Echut and Kusubo) is the main vector with the agreement of *W* = 0.9(*p* < 0.05). Perception on human trypanosomosis varies between Benna Tsemay and Gnagatom districts. Therefore, further study supported by laboratory like molecular test is very important to conclude the presence of human trypanosomosis in the suggested area. The overall prevalence of cattle trypanosomosis was 10.1%. The prevalence of trypanosomosis was significantly higher in poor body condition (OR = 2.1, *P* < 0.05) and in black coat color (OR = 13.5, *P* < 0.05) animals. *T. congolense* and *T. vivax* were circulating in the area. A total of 455 *Glossina* (385 *G. pallidipes*, 17 *G. tachinoides*, and 53 *G. fuscipes*) were trapped. The overall apparent density of *Glossina* was 3.79 Flies/Trap/Day. Three species of Glossina, namely *G. pallidipes, G. tachinoides*, and *G. fuscipes*, were distributed in the study areas. Therefore, the finding suggests that the problem is significant and the human trypanosomosis is doubtful. Hence regular control measures and molecular diagnosis need to be conducted.

## 1. Introduction

Animal and human trypanosomosis is one of main health constraints in sub-Saharan African countries, which affect about 37 countries in African continents. The disease transmission mainly depends on tsetse flies' distribution in African continents, where the distribution ranges between the latitude of 14° N and 29° S, South of the Sahel desert, North of Namibian, and Kalahari deserts [1]. Among biologically transmitted trypanosome species *Trypanosoma brucei gambiense* and *T. b. rhodesiense* are responsible for human trypanosomosis, whereas *T.b. brucei*, *T. congolense*, and *T. vivax* are causative agents for animal trypanosomosis [2]. The backbone of agriculture is significantly affected due to the economic impact of animal trypanosomosis; it reduces the cattle population by 37–70% and meat and milk production by 50% and reduces the draught power by 37% [3]. Human trypanosomosis is also a debilitating and fatal disease. Activities like tourism, hunting, honey collection, and herdsmen that are moving to game reserve and national park are considered as at high risk [1]. The occurrence of HAT to new area has been attributed to war, migration [4] of carrier populations from active foci, environmental deterioration, increasing parasite drug resistance [5], changes of the tsetse flies host preference, genetic variability of the parasite, and the existence of asymptomatic parasite-infected individuals.

Pastoral community in Gnangatom and Benna Tsemay districts depends on livestock production. The lifestyle is relay on herding their animals to feed and watering. Working age group of Gnangatom community moves up to South Sudan to the place called Nayta during dry season and will come back to their original village during the rainy season. Benna Tsemay communities experienced in moving up to Mago National Park, which is within Ethiopian territory. River Mago and the national wildlife reserve have given them a good opportunity to use as an alternative source of feed when there is feed scarcity. Honey collection and hunting are also practiced within the reserve site. Despite all activities in the border South Sudan and national park, there was no study conducted about community perception of human African trypanosomosis in the area. The retrospective study conducted in the Gambella region (Western Ethiopia) revealed that there has been HAT case report since 1976 to 1991 in Gambella region in Gilo Health Center and Gambella Hospital [6]. Community participatory investigation plays a main role in extrapolating the problem in a given area [7, 8]. The method was applied using interview, ranking, scoring, and visualization [9]. Hence, the study was carried with the objectives to understand community perception on major livestock diseases including bovine trypanosomosis, assess perception of human trypanosomosis, and study trypanosomosis prevalence and apparent vector density in the selected area.

## 2. Materials and Methods

### 2.1. Study Area

**Figure 1:**
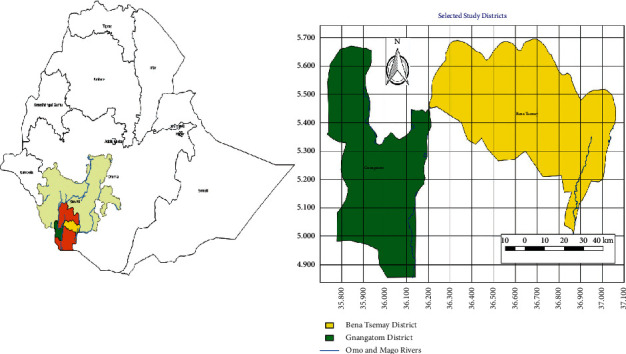
Map of Gnangatom and Benna Tsemay districts.

The study was conducted in Gnangatom and Benna Tsemay districts of South Omo zone, between April 2019 and March 2020. The study area is located in Southern Nations, Nationalities, and People Regional State (SNNPRS) of Ethiopia in a geographical position within the gridline of 04.90 to 5.60^0^ N and 35.80 to 36.90^0^ E as indicated in Figure 1. The Gnangatom district is located at the bordering of South Sudan and North West Kenya. Due to free movements, pastoralists move to either of the two countries with their cattle depending on the weather situation in search of feed and water. On the contrary, Benna Tsemay district is located 40 km away from the capital town of South Omo zone “Jinka”; it extends to the eastern direction up to Woyto River, and from the South West direction, it reaches up to Mago National Park. Pastoralists in this district move within the district and sometimes into Mago National Park. They do not have access to move outside the Ethiopian Territory further to south because of the tribal conflict between Hammar and Gnangatom tribes. The average annual rainfall in these two districts is erratic and it ranges from 700 to 1000. Usually, between December and April, the rainfall is excess. The mean annual temperature is 20.4°C. The altitude in Gnangatom ranges from 375 to 420 m.a.s.l., while in Benna Tsemay it ranges from 1100 to 1600 m.a.s.l.

**Figure 2:**
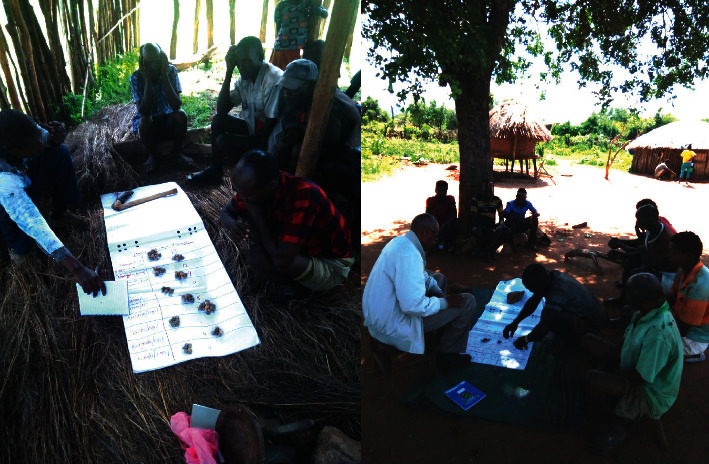
Active participation of group members.

Purposive snowball sampling was used to identify a representative of key informants; accordingly, 12 focus group discussions are shown in Figure 2, conducted in both districts to understand the community perception. Six representative pastoral associations in each district were selected as shown in Table 1, where pastoral association (PA) is the lowest government administrative structure, namely:

### 2.2. Data Collection Methods

#### 2.2.1. Participatory Epidemiology (PE) Tools

Participatory epidemiology (PE) tools were used as described by Catley [7, 10]. PE tools included proportional piling, matrix scoring, and seasonality. Stones were used as counters for scoring and ranking purposes. Each group discussant took 2 to 3 hours depending on the level of understanding of the group. Only key informants who have shown willingness to participate and contribute in the discussion were allowed to participate in the investigation. Facilitators were attentively monitoring the discussion to ensure that every group member had an equal chance to reflect his/her opinion to avoid dominance of the discussion by few participants. At the end of the discussion, stones were used as means of reflecting opinion agreement of that group and this was counted and recorded. Three facilitators “lead facilitator (researcher), assistant facilitator (translator), and one recorder” were involved during participatory investigation. Training was given to facilitators especially for recorders and assistant facilitators, prior to starting the study. Modeling all PE procedures was conducted in pretested one nonselected village to familiarize facilitators with the activities. The data collected in these pretest activities were not included in the main data.

#### 2.2.2. Proportional Piling

Proportional piling was used to estimate the relative livestock population in the area and the most important cattle diseases encountered. At the beginning of the discussion, the researcher allowed the participants to list the types of livestock kept in the area. Then a pile of 100 small and equal sized stones was given to group members in order to distribute them proportionally according to the number of animals existing in their village, where a large proportion of stones piled for large size and small proportion for small size livestock species.

Following livestock population ranking, participants were asked to list the most important cattle disease and allowed to pile proportionally [10] according to severity and/or occurrence of the diseases. High proportion of stone pile means that the disease in cattle was more important in terms of severity; and impacts on productivity, mortality rate, drug cost, or year-round prevalence. Every informant was asked to remove or add the already allotted stones to indicate the relative proportion of cattle disease until all participants agreed upon allocated proportion. A semistructured interview (SSI) was also used to support better comparisons among the diseases. This activity was repeated in all twelve focus groups of the two districts. Each group was composed of 6 to 15 on average of 10 participants according to their availability and interest to participate in the discussion.

#### 2.2.3. Matrix Scoring

The matrix scoring method was used to understand the community perception about the top listed diseases including clinical signs, cause of disease, seasonality, and age variability as described by Catley [7, 10]. To verify how well they are responding to indicators of each disease, diseases that were commonly known by facilitators and participants were designated as control and used for cross-checking [11] as described by Catley et al.[10]. Simple matrices were constructed to associate diseases against clinical signs and other epidemiological features (indicators). Disease names were represented using known material pictures, which were placed along the top *X*-axis of the matrix, while their indicators were placed along the left *Y*-axis. The responses were compared with standard veterinary medical textbooks [12]. Group discussants showed the relationship between each indicator against the list of diseases using piles of 30 stones. During the scoring process, informants were allowed sufficient time to discuss, and when necessary, probing questions were also posed in order to clarify the information. The level of agreement across the groups was analyzed using Kendall's coefficient concordance [13].

### 2.3. Parasitological Studies of Bovine Trypanosomosis

To estimate the prevalence of bovine trypanosomosis in the selected area, conventional field diagnostic materials and techniques are employed.

#### 2.3.1. Study Design and Site Selection

A cross-sectional study design was conducted from April 2019 to March 2020. Cattle herd from the selected Kebele were brought together, and hence the sampling was conducted using a systematic random sampling technique.

#### 2.3.2. Sample Size and Sampling Strategy

For the purpose of sample collection and due to communal grazing system in the area, cattle population in the selected Kebele was considered as one herd. A total of 288 cattle were bled from two districts, and 144 animals were sampled from each district. The systematic sampling method was used until the sample size attained. To compute the sample size needed for the study, a prevalence report of 7.8% (Abebe *et al.*, 2017) [14], 95% confidence interval, and 5% desired absolute precision were considered. Then a formula given by Thrusfield (2018) [15] was used for a simple random sampling technique. One hundred eleven sample sizes were determined, but to widen the opportunity of getting reliable information nearly 60% sample size increased. Accordingly, a total of two hundred eighty-eight animal samples were bled, because of the aggressive nature of the animals and handling problem of the herdsmen stopped handling, and hence, it was difficult to attain more than 60% added. The age of selected animals was estimated by dentition and the body condition status of the animals was assessed on the criteria described by Nicholson and Butterworth (1986) [16].

Despite nine classifications, a body condition score was categorized into poor, moderate, and good body condition scores in this study. The animals showing emaciation, visible ribs, pointed dorsal spine, prominent transverse process, visible hip bone, and tail head were considered as poor, animals with less visible ribs, smooth, and well-covered categorized as moderate, and animals with heavy deposit fat clearly visible on tail, head, ribs, brisket, and dorsal spine were considered as good body condition score.

#### 2.3.3. Blood Collection and Parasitological Examination

Bleeding of selected animals was conducted using bleeding lancet and heparinized capillary tube. Meanwhile, information of each selected animals is recorded like coat color, sex, body condition score, and age. Then blood was centrifuged in 12,000 rpm using a hematocrit centrifuge for 5 minutes at field site and was removed after centrifugation. Pack cell volume (PCV) was measured by an HCT reader and the results were recorded to estimate anemia. The buffy coat/plasma junction with RBC was examined by cutting 1 mm below buffy coat to include the upper most red blood cells; droplet expressed on a clean microscope slide and then covered with a coverslip and a wet film examined under 40x objective magnifications using dark-ground or phase-contrast illumination to detect motile trypanosome parasite (Murray *et al.*, 1977) [17]. Species of trypanosome were confirmed by a thin smear stained with Giemsa and examined under100x objective magnification.

### 2.4. Entomological Survey

The entomological survey was conducted in selected districts from April 2019 to March 2020. A total of 60 NGU traps, baited with acetone and fermented cow urine, were deployed near to watering and grazing site closer to shelter trees and bush, which were commonly visited by animals. All the traps were deployed at about 200m air distance intervals for 48 hr within the altitude of 388 to 1100 m.a.s.l. in the geographical location of 5.41′22″ N and 35.69′65″E; 5.17′82″ N and 36.07′ 52″ E. Then after, the flies were collected from the traps and counted, and sex and Glossina species were identified following the standard procedure (Uilenberg, 1998; Pollock, 1982) [18, 19]. Other caught biting flies were identified at the genera level according to their morphological characteristics such as size, color, wing venation structure, and proboscis.

### 2.5. Data Management

Qualitative data collected from the group discussion were entered into the Microsoft Excel spreadsheet. The data were then exported to Statistical Package for Social Sciences (SPSS®) base 20 (Inc., Chicago, IL, USA) for the statistical analysis. The Kendall's coefficient of concordance (W) was used to determine the level of agreement among groups [20]. W (Kendall's coefficient of concordance) ranges from 0 to 1. The value of “W” closer to 1 is the higher agreement among the informants. According to the critical values for Kendall's coefficient concordance (W), agreement was termed as weak, moderate, and good if *W* = values were less than 0.26, between 0.26 and 0.38 (*p* < 0.05), and greater than 0.38 (*p* < 0.01 to <0.001), respectively. Parasitological and entomological studies are analyzed using SATA software.

## 3. Results

### 3.1. Community Perception

#### 3.1.1. Major Livestock Production

Livestock production is the livelihood of the communities in two districts. Proportional piling showed that the cattle population scored the highest followed by goats and sheep with the median score of 32(14–40), 26(12–33), and 21(5–23), respectively, as shown in Table 2. Donkey population is significantly high in Gnangatom communities than in Benna communities. Donkey in this community is used not only for pack animals but also for festivities. Cattle, goats, and sheep were all local breeds of zebu, konso-woyto, and Somali black head, respectively. There was no camel rearing experience. Most of Gnangatom herdsmen move with their cattle as far as South Sudan to the place called Nayta and will come back to their village during a rainy season. Usually, goats and sheep are kept around their village, whereas Benna herdsmen move as far as Mago National Park in search of feed and water. Herdsmen complained that animals often infected with trypanosomosis in the national park because there is high population of buffalo fly “tsetse fly.”

#### 3.1.2. Major Cattle Diseases

Diseases mentioned by participants were translated by local veterinarian who have worked for longtime in the area and created agreement between local name and scientific name of the disease which was commonly used in the field. According to the two districts community informants, major cattle disease that affects animals in the area ranked in Table 3. Trypanosomosis and contagious bovine pleuropneumonia were ranked as first and second, with proportional median score of 24(13–26) and 23(19–26), accordingly. In both two communities unanimously mentioned that the effect of trypanosomosis is very severe because of its negative socioeconomic impact. Blackleg and anthrax in the third position and circling disease were in the last position. Circling disease was not specific type of diseases but they have mentioned that there is circling disease occurrence periodically within a year and communities were unable to treat the case traditionally.

### 3.2. Disease Signs Listed and Agreement with Trypanosomosis

**Figure 3:**
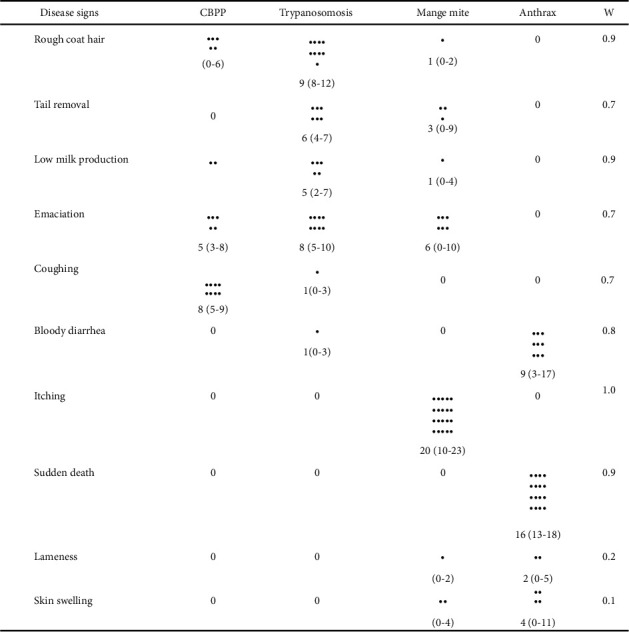
Matrix scoring of disease signs against diseases, *n* = 12 group participants, black dots are median score, and ranges are in parenthesis; *W*=Kendall's coefficient concordance test of agreement.

Community perceptions on signs of diseases were analyzed using a matrix scoring technique in selected diseases, trypanosomosis being a center of focus. The most frequently mentioned and the first four ranked diseases were subjected to matrix scoring. CBPP, Trypanosomosis, Anthrax, and mange mite were placed on top of the *x*-axis. Signs or indicators are placed along the left side of the *y*-axis. Twelve group participants from both Gnangatom and Benna elders completed the matrices without problem. A high median score of disease signs is an indication of the main sign of a given disease. The analysis of disease signs against each disease demonstrated a good agreement among group discussants for eight disease signs as shown in Figure 3. There was poor agreement among the informants on two disease signs “Lameness and Skin swell” among diseases listed. During discussion, participants provided information about different stages of trypanosomosis. Acute trypanosomosis indicated a rough coat hair, whereas in the chronic case, tail removal and emaciation exhibited. There was a strong agreement between itching and mange mite, whereas bloody diarrhea and sudden death were for anthrax. Coughing was strongly associated with CBPP. Informants explained about anthrax sudden killing characteristics and agreed that consuming the carcass suspected with anthrax case is dangerous to human. Even they said human can contract the disease from the hide died of anthrax. In the previous period, communities have been eating and getting sick, because of the strong health extension work these days they do not consume, indicating that they have been eating animals died of any cases.

### 3.3. Cause of Disease Transmission

**Figure 4:**
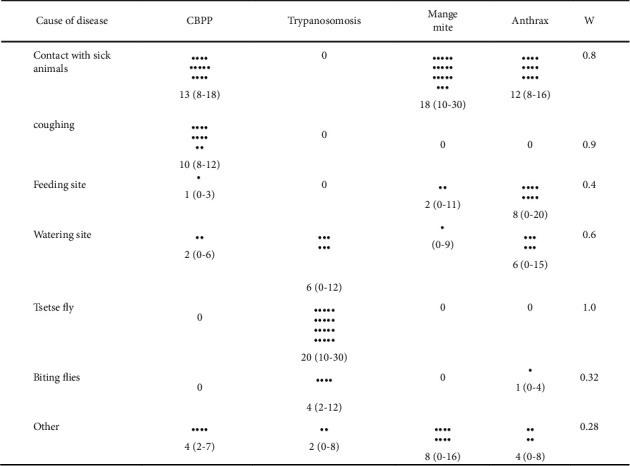
Summary of cause of disease by 12 group participants (each group with 6–15 participants): black dots are median score and ranges are within parenthesis, *W*=Kendall's coefficient concordance test of agreement.

Informant's reflection was cross checked by action and demonstration of specimens. Participants agreed unanimously that animal contact with diseased animals and coughing were the main cause of CBPP disease transmission. Similarly, feeding and watering site were also demonstrated and reached consensus, whereas specimens were used to identify tsetse and biting flies to reach with common understanding of their reflections. Tsetse fly locally called “Echut” by Gnangatom and they strongly believe that it is a cause of trypanosomosis locally called “Lokurion” whereas Benna community named tsetse “Kusubo” which is also the name for disease trypanosomosis. Both communities strongly agreed that tsetse is the cause of trypanosomosis. They explained that cattle are infected especially at watering site where tsetse flies are abundant and their close contact to the buffalo will intensify the tsetse bite when animals move to Mago National Park. They strongly believe that tsetse fly is a buffalo fly biting flies other than tsetse, which were also mentioned as a cause of trypanosomosis, but it was with moderate agreement (Figure 4). Both community participants strongly agreed that the cause of CBPP was through contact and coughing, and mange mite was caused through direct contact with mange infected animals. Animals grazing in place where animals previously dead with anthrax and animals licking anthrax infected hide were considered as source of infection.

### 3.4. Seasonality

**Figure 5:**
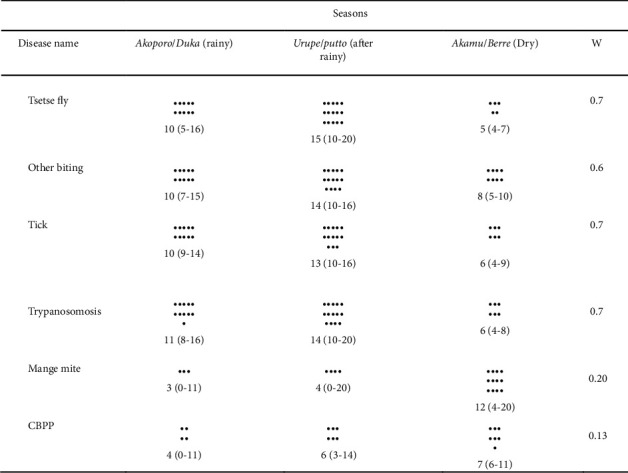
Seasonal matrix score summary; words of Gnangatom/Benna are in *italics*, respectively.

Three seasons were mentioned by both Gnangatom and Benna community elders. A rainy season is considered at any time when there is rain, which does not follow months of the years. A rainy season varies from year to year. Sometimes rain starts at February and ends at June, sometimes it stops by April, and sometimes it extends up to September. A rainy season is called as Akoporo and Duka by Gnangatom and Benna. A dry season is also influenced by the presence or absence of rain. So, there is no constant month. Akamu or Berre (Bonna) is a local name for a dry season by respective community. Shortly after rainy season, it is called as Urupe or Putto by Gnangatom and Benna discussants, respectively. After rainy season tsetse fly population is high according to the discussants. There was a strong agreement among group discussants about seasonality of tsetse fly, biting flies, trypanosomosis, and tick abundance (*W* > 0.38),whereas it was observed that the agreement was poor among groups about the seasonality of CBPP and mange mite (*W* < 0.26) as shown in Figure 5.

### 3.5. Community Perception on Human Trypanosomosis

All groups in Benna community unanimously agreed that tsetse flies bite herdsmen and cause small bleeding, irritation, and temporary local skin swelling. This swelling will regress to the normal within half an hour. They do not have the experience of human disease associated with tsetse fly bite. Benna community travels as far as Mago National Park where the tsetse fly catches per trap per day reaching more than 50 flies. They usually understand malaria cases and treat with modern treatment. Communities are not suspecting human trypanosomosis, even they do not have awareness that tsetse can transmit human trypanosomosis, despite their rich knowledge about animal trypanosomosis about its symptom, seasonality, and cause of disease transmission.

Gnangatom community elders also agreed that tsetse is a notorious fly that bites nervously and causes bleeding and irritation as well as small swelling. They have no idea about the human disease related to tsetse bite. However, among participants, few of them were responded during probing questions about the presence of human trypanosomosis due to tsetse bite especially in an area called Nayta within South Sudan territory. The local name of the disease called “lokoyt.” It is a chronic disease; the infected man suffers with wasting body weight, debilitation, itching or pruritus, and small nodules throughout the skin. The sign mentioned was in agreement with the literature and books [1, 12, 21]. After long-time debilitation if there is no treatment, the patient will die. This kind of disease is common in South Sudan at Nayta district according to the informants. A pastoralist at Gnangatom district experiences such kind of case because herdsmen move freely with their animals up to Nayta South Sudan, to place where the disease is suspected and stay long until they come back. Group participants in study villages were asked their understanding and the action at the onset of febrile condition, malaria was typically suspected, and antimalarial drugs medication started, which is available in the public health post. If the sign remains persistent, they will continue antimalaria drug treatment, which can mislead the diagnosis of other related diseases like human trypanosomosis.

Information was collected from health workers at Jinka Zonal Hospital, Benna Tsemay, and Gnangatom Health Center. They have theoretical knowledge about sleeping sickness, symptom, and cause of the disease. All of them did not come across with such type of cases other than malaria and yellow fever in the area. Laboratory diagnosis usually focuses toward malaria. Pharmacist and drug dispensary workers have no drugs related to human trypanosomosis case.

### 3.6. Exposure to Risk Factor of Human Trypanosomosis

Majority of the respondents were pastoral communities who live bare skin except shorts and were exposed for direct tsetse bite and/or can acquire the disease human trypanosomosis infection. Herdsmen usually move through a wide forest area with their cattle for grazing, to the River side for watering, bath, or cloth washes or water fetches. The elder among communities remain at home, and civil servants and health professionals are less exposed to direct contact to the tsetse flies. All respondents unanimously know tsetse fly and they strongly believe it transmits to animal trypanosomosis but less known about human trypanosomosis.

### 3.7. Triangulation (Crosschecking)

To create common understanding between researcher and community response, their respective veterinary clinic record was observed. Accordingly, the major disease problem in the area as far as cattle concerned was trypanosomosis, followed by disease outbreak of blackleg and anthrax. Lumpy skin disease (LSD) and CBPP are also reported. However, due to the strong support by southern pastoral development program (SPDP), sufficient vaccination conducted for disease outbreaks yearly. The problem of trypanosomosis was attempted to overcome by a regular treatment program, though it was not covered to the level of demand. Human health center was used to collect information about human trypanosomosis disease and related drugs. Professionals were asked at different hierarchical positions, who were assigned at health post, district, and zonal hospital level about human trypanosomosis. They did not come across with such type of cases and they do not have a certain type of drugs supposed to treat this case.

#### 3.7.1. Trypanosomosis Prevalence

Out of 288 cattle bled for parasitological and hematological study, 29(10.1%) of them found positive for trypanosomosis infection. Univariable and multivariable logistic regression analysis results of potential risk factors considered for the occurrence of trypanosomosis in this study are shown in Table 4. After the risk factor analysis with univariable logistic analysis, those variables with *P* < 0.25 were further subjected to multivariable analysis. Only poor body condition score and black color animals showed significantly (<0.05) high prevalence.

#### 3.7.2. Trypanosome Species Identified

Two species of Trypanosomes were identified, which are in order of abundance *Trypanosoma congolense* (79.3%) and *Trypanosoma vivax* (13.8%). About 6.9% of the animals were infected by mixed Trypanosome species, and *T. congolense* and *T. vivax* were found in both the study areas as shown in Table 5.

#### 3.7.3. Hematological Findings

The overall mean PCV value of all studied animals was 23.8%, which was in the anemic state, and the detailed result is indicated in Table 6.

#### 3.7.4. Entomological Findings

A total of 455 *Glossina* species and 689 biting flies were caught with 60 NGU traps deployed in the study areas. The overall apparent densities of *Glossina* species and biting flies were 3.79 F/T/D and 5.74 F/T/D, respectively (Table 7). In general, three species identified, of these *Glossina tachinoides* and *Glossina pallidipes* were recorded in Gnangatom, whereas *Glossina fuscipes* and *Glossina pallidipes* are recovered in Benna Tsemay district. The sex proportion of *Glossina tachinoides* was 41% male and 59% female, that of *Glossina fuscipes* was 28.3% male and 71.7% female, and the sex proportion in *Glossina pallidipes* was 28.3% male and 71.7% female recorded. The biting flies that are commonly encountered were *Stomoxys* species (61.2%) and *Tabanus* species (38.8%).

**Figure 6:**
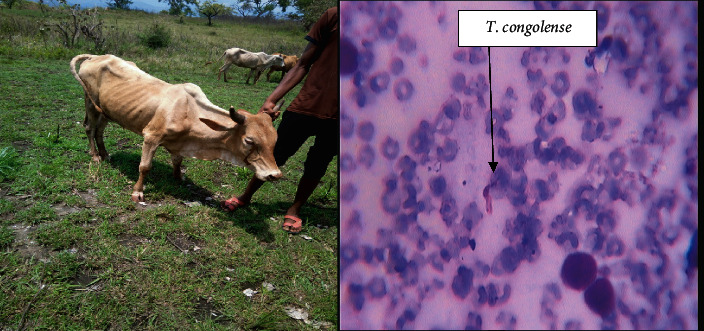
Animal identified by participants infected with trypanosomosis and Giemsa stain.

Trypanosomosis-suspected animals were selected by participants and bleed. The smear stained with Giemsa stain picture is shown in Figure 6. They showed that 8.3% (8/96) prevalence of trypanosomosis observed. Most of the animals selected had typical clinical signs; this finding supported the skill of the community how well they were describing the clinical signs of major diseases in their locality.

## 4. Discussion

Livestock production and their major health constraints were studied to understand community perception on animal and human trypanosomosis in the area. This investigation provided baseline data for further extension intervention to improve health status through appropriate control methods and additional research ambiguous issues.

### 4.1. Disease Knowledge

This study focused on animal and human trypanosomosis to understand community knowledge and their experience of identifying disease clinical signs, cause of transmission, and disease seasonality. Hence, a similar participatory rural appraisal (PRA) technique was applied in order to investigate community perception on human trypanosomosis in their locality [7].

Among major cattle diseases listed by both districts, the overall median score indicates that bovine trypanosomosis ranked first followed by CBPP. Pastoralist agreed that trypanosomosis is the main health problem of their cattle. Participants were good in recognizing the clinical signs of bovine trypanosomosis. Rough coat hair, low milk production weakness at early stage, and emaciation and tail removal at the chronic stage of trypanosomosis were the clinical signs strongly agreed by participants. Knowledge of the participants was cross checked by asking clinical signs of anthrax, CBPP, and Mange mites; therefore, bloody diarrhea and sudden death for anthrax, coughing for CBPP, and intense itching for mange mite were responded and the agreement among groups were strong. Focus group discussion seems contributed much for correct diagnosis because during discussion every participant idea enriched before the final allocation of stones for each clinical sign. Majority of the clinical signs observed were consistent with accepted veterinary knowledge written in literatures and reference books [12, 22].

Participants were also identified a cause of transmission trypanosomosis. They underline that the main cause of bovine trypanosomosis is tsetse bite followed by watering site. Watering site was associated with the abundance of tsetse at river side. The agreement among group was good (*W* > 0.38). This concept agrees with Ref. [23], and tsetse flies prefer moisturized and favorable humidity than dry environment. Tsetse and trypanosomosis are a treat to cattle production mainly in the main river basin such as Omo, Baro, Didessa, Abay, and their tributaries in Ethiopia [24]. Other biting flies and “other” (sick animal, feed shortage) could cause a trypanosomosis disease, but the agreement among groups was moderate (*W* < 0.38 and >0.25). Pastoralist strongly agreed that disease CBPP, anthrax, and Mange mite are caused by a direct contact with sick animals. During probing question participants further explained that CBPP transmission occurs through cough, whereas animals get infected with anthrax when they graze in the previously died field, especially at the rainy season when green grasses are available. They have also mentioned that the hide of anthrax dead animals could also cause the disease. This knowledge is in agreement with accepted veterinary reference books [8, 12].

Participants observed the seasonality of tsetse fly, other biting flies, tick infestation, bovine trypanosomosis, Mange mite, and CBPP. Tsetse fly, tick, and trypanosomosis are more abundant during rainy and immediately after the rainy season; the response among groups was strongly in agreement using Kendall's coefficient concordance (*W* = 0.7). A similar observation by the Tanzanian pastoralist has shown that the rainfall and temperature were important factors for the abundance of tsetse fly and tick population [25], where following rainy season the weather is favourable for reproduction of tsetse flies and tick population [26]. Other studies also demonstrated the influence of rainfall and temperature on tick and tsetse ecology [27, 28]. Similar observation was recorded by Seyoum et al. 2013 [29] in the rainy season; despite the presence of feed and well-feed animals, fly challenge and trypanosomosis were very high in rainy and immediately after rainy season. A significant trypanosome burden was also observed in the rainy season [30].

### 4.2. Impact of Bovine Trypanosomosis

The community livelihood depends on livestock production, and the cattle production takes the leading position to determine their social status as well as wealth. However, according to participants, adult cattle are the most chronically affected animal groups by trypanosomosis, which is in agreement with records of 37–70% that results on reduction of meat and milk production by 50% [3]. This finding was supported by Catley et al. [31] in Tana River district of Kenya, by Adam et al. [32] in Ghana, and by Von Wissmann et al. [33] in west Kenya. Abortion is common, cattle will not be marketable, animals lose body weight, and repeated trypanocidal treatment is costly. The use of cattle for dowry and barter severely affected due to weight loss and emaciation as result of trypanosomosis infection. Poor growth and death are also reported [22, 34]. Tesfaye et al. [30] observed that 50.9% of respondent claimed draught power reduction and drug cost was the main impact of trypanosomosis in their village. This rich experience of pastoral community to make correct diagnoses suggests that their participation is paramount important to identify and prioritize local issues in question in general and livestock health issues in particular.

### 4.3. Human Trypanosomosis

Knowledge of animal trypanosomosis was aligned with reference books and showed their rich endogenous knowledge as compared to poor knowledge on human trypanosomosis, despite communities were near to game reserve area and bordering South Sudan. Herders' movement across the border into the Human African Trypanosomosis (HAT)-suspected area is common. The chance of tsetse bite is very high. Few participants among focus groups in Gnangatom tried to explain the presence of HAT, which is common in South Sudan. This should not be overlooked, since there is a continuous movement in and out in this border area. Chronic and sporadic as well as nonspecific symptom characteristic nature of the disease may be the reason for poor recognition and or may be due to trypanotolerance nature of individual after being infected [35]. The lack of good knowledge could be due to nonendemicity of the disease in the reserve [36]. Whereas in an area where HAT is endemic, community respondent put HAT as the priority disease in their village because the infected person has a long recovery period and even will not recover to the previous productive stage [21].

### 4.4. Animal Trypanosomosis Prevalence and Vector Apparent Density

In general, the overall trypanosomosis prevalence (10.1%) was in agreement with previous reports in nearby zones [14]. The prevalence of bovine trypanosomosis in the southern part of the country was ranging from 1.3% to 29.5% [37, 38], using buffy coat methods. The finding shows that there was no significant difference between two districts and among Kebeles where samples were collected. This might be due to environmental and livestock keeping similarity because both communities are pastoralist and they are adjacent districts. The multivariable analysis revealed that significantly a higher prevalence was recorded in the poor body condition (*p* < 0.05, OR = 2.1) and black animals (*p* < 0.05, OR = 13.5). Due to chronic nature of the disease, majority of the sampled animals had a poor body condition score and had significantly high prevalence than medium and good body condition scores, even though the poor body condition could exist with other risk factors. Black color animals more affected by trypanosomosis than other colors. This might be the reason that majority of animal colors were different from black. Such a significant result was also observed by other works [14, 39]. Scholars indicated that tsetse flies are highly attracted by blue color from the distance and are usually alighted on black colors [23].

More prevalently observed *T. congolense* in this study was in agreement with other works [14, 39]. The reason for uncertain despite *T. vivax* transmission could exist in both cyclical and mechanical means.

The mean PCV (21.9%) of infected animals was significantly lower (*t* = 2.38, *P* < 0.05) than the noninfected animals. Trypanosomosis-infected animals reduce its PCV regardless of feed quality provided for the infected animals [22]. On the other hand, nutritional deficiencies are also known to reduce PCV [12]. Although trypanosomosis is characterized by a reduction in PCV, other factors like parasitism also cause a reduction in PCV.

Three species of Glossina were identified, namely, *Glossina tachinoides*, *Glossina pallidipes*, and *Glossina fuscipes*. But in each study district, only two species recovered where *Glossina pallidipes* was common for both study sites. These species were also reported by various authors from areas neighboring to the study district in a different period [14, 38, 39]. The overall apparent density of Glossina species caught during the study period was 3.79 F/T/D. This is an indication that there is tsetse fly abundance in the study area. Riverine and savanna type of tsetse in the area indicates animals at watering as well as at grazing site, which has a risk of tsetse bite and then disease transmission.

## 5. Conclusion

Community perception in the study area has indicated that there is rich experience in describing livestock production and major livestock diseases. In this assessment it was understood that bovine trypanosomosis and tsetse fly, which is the cause of the disease, is a priority disease problem where they need support. The disease confirmed clinically as well as using laboratory. Human trypanosomosis poorly understood by participants may be due to low endemicity and disease tolerance. Despite favorable condition existed for the occurrence of HAT in the area. The overall prevalence of trypanosomosis in cattle was 10.1%. Two species of trypanosoma were circulating in the study area, *T. congolense* and *T. vivax*, with significantly high *T. congolense* proportion. The prevalence of trypanosomosis was significantly higher in poor body condition, and black coat animals. Relatively high apparent density of tsetse flies caught which shows the importance of the problem. Three species of Glossina namely *G. pallidipes*, *G. tachinoides* and *G. fuscipes* were distributed in the study areas.

## Figures and Tables

**Table 1:** List of pastoral association selected with its respective participants.

s/n	Gnangatom PAs	No. of participants	Benna Tsemay PAs	No. of participants
1	Kakuta	15	Gurma mero	10
2	Narogoye	7	Goldia	9
3	Napusemiria	6	Stimba	14
4	Kibish	12	Dizi	6
5	Refugee camp	8	Baytsemal	7
6	Lokorilem	10	Kure	12

**Table 2:** Major livestock kept and their proportional rank in Gnangatom and Benna districts.

Common name	Gnangatom local name	Median and range *n* = 6	Benna local name	Median and range *n* = 6	Proportional rank
Cattle	*Natuk*	32(30–33)	*Waki*	31(14–40)	1^st^
Goat	*Nammae*	26(25–26)	*Quela*	26(12–33)	2^nd^
Donkey	*Esikiria*	14(12–25)	*Uquoli*	7(3–10)	4^th^
Sheep	*Hamesekin*	20(8–24)	*Yati*	21(5–23)	3^rd^
Chicken	*KoKora*	8(6–10)	*Bacha*	15(4–17)	5^th^

**Table 3:** Major cattle diseases according to Gnangatom and Benna communities and their respective proportional piling of median and range score.

Common name	Gnangatom	Median and range ^*∗*^*n* = 6	Benna Tsemay	Median and range ^*∗*^*n* = 6	Rank
CBPP	*Lowkogne*	23(19–26)	*Shompo*	19.5(10–22)	2^nd^
Blackleg	*Lotom*	15(13–15)	*Narka*	9(4–12)	3^rd^
Trypanosomosis	*Lokurion*	22(21–24)	*Kusubo*	24(13–26)	1^st^
Anthrax	*Longole*	17(12–19)	*Chishe*	7(4–14)	3^rd^
Mange mite	*Lomeri*	7(6–10)	*Katsi*	8.5(6–14)	5^th^
Ticks	*Umadhan*	4(3–6)	*Shoquo*	9.5(6–15)	6^th^
Circling death	*Malere*	7(2–7)	*Chirchia*	4(0–13)	7^th^
FMD	*Lokojen*	8(3–10)	*Shokolo*	6(1–8)	5^th^

^*∗*^ *n* = number of groups used in each district, in each group 6–15 participants involved. Words of Gnangatom and Benna in *italics,* and ranges are in the parentheses.

**Table 4:** Univariable and multivariable logistic regression analysis of trypanosomosis infection in relation to factors.

Risk factor	n	+ve	Prevalence (95% CI)	Univariable			Multivariable		
				OR	95%CI	*p* value	OR	95% CI	*p* value
*Woreda*									
Gnangatom	144	10	6.9(3.6,12.7)	Ref	-	-	Ref	-	-
Bennatsemay	144	19	13.2(7.2,19.1)	2.04	0.91–4.5	0.083	1.9	0.8–4.3	0.122
*Kebele*									
Goldia	48	3	6.2(1.6, 18.2)	Ref	-	-	-	-	-
Dizi	48	5	10.4(3.9,23.4)	1.7	0.4–7.8	0.465	-	-	-
Kure	48	2	4.9(0.7,15.4)	0.9	0.2–4.10	0.648	-	-	-
Narogoye	48	5	10.4(3.9,23.4)	1.7	0.4–7.8	0.465	-	-	-
Kibish	48	6	12.5(5.19,25.9)	2.1	0.5–9.11	0.302	-	-	-
Kakuta	48	8	16.7(7.97,30.8)	3	0.7–12.1	0.122	-	-	-
*BCS*									
Good	16	1	6.3(0.3,32.3)	Ref	-	-	Ref	-	-
Median	101	5	4.9(1.8,11.7)	1.4	0.4–6.5	0.261	1.4	0.4–6.51	0.261
Poor	171	23	13.5(8.8, 19.5)	2.1	1.13–13.1	0.031	2.1	1.13–13.1	0.040
*Age*									
≤2 yrs	14	1	7.0(0.4, 35.8)	Ref	-	-	-	-	-
2 ≤ 6 yrs	107	15	14.0(8.3,22.4)	2.1	0.3–17.4	0.481	-	-	-
>6 yrs	167	13	7.8(4.4, 13.2)	1.1	0.13–9.1	0.981	-	-	-
*Sex*									
Female	190	21	11.1(7.1,16.6)	Ref	-	-	-	-	-
Male	98	8	8.2(3.9,15.9)	1.4	0.6–3.3	0.442	-	-	-
*Color*									
White and spotted	69	5	7.2(2.7, 16.8)	Ref	-	-	-	-	-
Red	160	15	9.4(5.5, 15.3)	3.92	0.9–17.6	0.461	2.78	0.8–14.7	0.483
Gray and roan	24	3	12.5(3.3,33.5)	5.73	1.1–7.4	0.055	5.73	0.9–17.4	0.061
Black and Brown	25	6	24(10.2, 45.5)	12.5	2.4–63.8	0.033	13.5	2.5–68.7	0.032

**Table 5:** Proportion of Trypanosome species identified in Gnangatom and Benna Tsemay districts (*n* = 29).

Districts	*T. congolense* (%)	*T. vivax* (%)	Mixed (%)	Overall
Gnangatom	9(31.0)	-	1(3.45)	10(34.5%)
Benna Tsemay	14(48.3)	4(13.8)	1(3.45)	19(65.5%)
Total	23(79.3)	4(13.8)	2(6.9)	29

**Table 6:** Mean packed cell volume in association with infected and noninfected animals.

Factor	No. examined	Mean PCV	SDV	95%CI	*t*-test	*p* value
Infected	29	21.9	±4.57	20.2–23.6		
Noninfected	259	24.03	±4.56	23.5–24.6	2.38	0.017
Gnangatom	144	22.98	±5.06	22.2–23.8		
Bennatsemay	144	24.65	±3.82	24.0–25.3	3.16	0.002

**Table 7:** Summary of the tsetse and biting flies count and apparent density in the study period.

Districts	No. of traps deployed	*G. tachinoides*	*G. fuscipes*	*G. pallidipes*	Total (F/T/D)	Biting flies (F/T/D)
Gnangatom	30	17(0.28)	-	45(0.70)	62(1.03)	597(9.95)
Bennatsemay	30	-	53(0.88)	340(5.67)	393(6.5)	92(1.53)
Total	60	17(0.14)	53(0.44)	385(3.20)	455(3.8)	689(5.74)

## Data Availability

The data used to support this study are available upon request.
